# Factors associated with the risk of suicidal behavior among adolescents transitioning to secondary school in Nairobi County, Kenya: a cross-sectional study

**DOI:** 10.11604/pamj.2022.43.180.35917

**Published:** 2022-12-07

**Authors:** Aggrey Gisiora Mokaya, Gideon Mutie Kikuvi, Joseph Mutai, Lincoln Imbugwa Khasakhala, Peter Memiah

**Affiliations:** 1 Center for Public Health Research, Kenya Medical Research Institute, Nairobi, Kenya,; 2 Department of Environmental Health and Disease Control, School of Public Health, Jomo Kenyatta University of Agriculture and Technology, Juja, Kenya,; 3 Department of Psychiatry, University of Nairobi, Nairobi, Kenya,; 4 Division of Epidemiology and Prevention, Institute of Human Virology, University of Maryland School of Medicine, Baltimore Maryland, USA

**Keywords:** Suicidal behavior, adolescence, Kenya, alcohol use, depression

## Abstract

**Introduction:**

adolescence is a transitory stage in the lives of young people. The transition from primary to secondary school among adolescents is associated with suicidal behavior but is not well characterized in the Kenyan context. This study sought to elucidate factors associated with the risk of suicidal behavior among adolescents aged 11-18 years in transition to secondary school.

**Methods:**

a cross-sectional design was employed in the study that was conducted among adolescents in 5 randomly selected secondary schools in Nairobi County. The study involved 539 students who had joined form 1 in January 2020. Data were collected using the suicide behavior questionnaire-revised (SBQ-R) in March 2020. Factors associated with suicidal behavior were assessed using a generalized linear model (GLM), using a poisson distribution with a log-link function to estimate adjusted prevalence ratios (aPR), and a significance level of p=.05.

**Results:**

one-fifth (20.04%) of adolescents with a median age of 14 years were at risk of suicidal behavior. Depression (aPR=3.16, C.I {1.85, 5.41}, p=0.001) and lifetime alcohol use (aPR=1.87, C.I {1.17, 2.97}, p=0.009) were found to be significant factors for suicidal behavior.

**Conclusion:**

depression and lifetime alcohol use are associated with the risk of suicidal behavior among adolescents transitioning from primary to secondary school. Interventions may need to be targeted at the pre-secondary or primary school level to prevent underage alcohol use and enhancement of social support to prevent depression in this demographic of the population.

## Introduction

Adolescence is a transitory stage in life: the transition from childhood to young adulthood, physical and morphological changes, and changes in social relationships. These transitions also coincide with the transition from primary to secondary school and this is likely to place an extra mental burden on adolescents. This is associated with a variety of challenges surrounding sexuality, rapid physical growth, interpersonal relationships, autonomy, and risk-taking behaviors among other issues. These issues can also predispose adolescents to suicidal behavior [1]. Adolescents account for over 250.000 annual deaths by suicide making suicide the second most prevalent cause of adolescent mortality around the world [2]. While studies on the prevalence and correlates of suicidal behavior are few in the Kenyan context, some authors have shown that suicidal behavior accounts for one in twenty deaths among children and adolescents in Kenya [3]. Depression, history of trauma, bullying, the development of social media and the platform it provides for cyberbullying, alcohol and substance use, parental rejection, and trauma are indicated for adolescent suicidal behavior. Other factors such as food insecurity and sexual intercourse are associated with an enhanced risk of suicidal behavior, especially among adolescent girls who are the most affected [4]. Indeed, a 2016 study demonstrated that adolescent girls have a higher likelihood of displaying suicidal behaviors. However, they are also less likely than boys to complete a suicide attempt [5]. The breadth of these associated factors points to a need for a context-specific understanding of suicidal behavior. This would help to inform contextually relevant suicide prevention screening and interventions [6-8]. While studies into adolescent suicidal behavior have tended to focus either on continuing students or a combination of transitioning and continuing students [9,10], we postulated that assessing adolescents in the transitory period would likely yield a deeper understanding of adolescent suicidal behavior at school. This has been demonstrated elsewhere, where adolescents transitioning to high school are at an increased risk of suicidal behavior [10]. The aim was to understand whether the predisposing factors for suicidal behavior were already present by the time adolescents were joining secondary schools or not. Guided by an intention to enrich the evidence base for adolescent mental health promotion in the school setting - where the opportunity to intervene exists [11]. This study sought to elucidate the factors associated with risk of suicidal behavior among adolescents transitioning between primary and secondary schools in Nairobi County, Kenya.

## Methods

**Study design and setting:** the study utilized a cross-sectional design. The study was carried out in 5 secondary schools in Nairobi County: 1 boy school (school A), 2 girls´ only schools (schools B and C), and 2 mixed schools (schools D and E). Of the 5, 3 were boarding schools (A, B, and C), and the rest (D and E) were day schools. Nairobi county is a cosmopolitan county in the heart of Kenya that hosts the capital city, Nairobi. It is an urban county and is the seat of political, economic, and social power in Kenya. By the time the study was being carried out (January to March 2020), there were a total of 71 public secondary schools in the county which were either mixed schools, boys- or girls-only schools. Some of the schools were day schools while others were boarding schools. The study data collection was done just before the first reported cases of coronavirus in Kenya.

**Study Population:** the study population comprised adolescents who had joined form 1 in the selected schools in January 2020. The selection criteria for the adolescents were that they would have to have been transitioning from primary to secondary school, would have attended the school for at least one month before data collection, and informed consent from the parents and assent from the adolescents themselves.

**Sampling:** the five schools were selected by simple random sampling from a pool of 71 schools. A convenience sample of 539 adolescents was recruited for the study based on meeting the study criteria. The sample size was arrived at using Fisher´s formula based on the prevalence of suicidal behavior of 27.1% [9], which gave a minimum sample size of 304. A design effect of 2 was applied to the sample size, and a total of 608 students were invited, consent and assent were received from 539 guardians and students who participated, and their data were analyzed.

**Data collection:** data were collected using a self-administered questionnaire in March 2020. Quality checks during data collection ensured that there was no missing data. The questionnaire collected data on adolescent sociodemographic characteristics (gender: binary either male or female, age: binary either 11-14 years or 15-18 years, description of caregiver: 4 categories mother only, father only, both or guardian); lifetime and past 30-day alcohol, drug, and tobacco use (binary: yes or no), socioeconomic status (classified as low 0-10, middle 11-20, and high 21+ based on an additive index of household ownership of various items), romantic relationship involvement (binary: yes or no), and sexual activity (binary: yes or no). Additionally, the suicide behavior questionnaire-revised (SBQ-R) was used to assess suicidal behavior, with an overall score of 7 or higher classifying an adolescent at-risk of suicidal behavior [12,13]. These questionnaires have been validated for use either in Kenya or other African countries for use with adolescent populations [9].

**Data analysis:** data analysis was carried out using STATA version 14. Descriptive statistics were computed as frequency distributions of the variables of interest in the study. The factors associated with suicidal behavior were analyzed through a generalized linear model (GLM), using a poisson distribution with a log-link function, to estimate adjusted prevalence ratios (aPR). This is because, for cross-sectional surveys, prevalence ratios are recommended as a measure of risk for common outcomes >10% compared to odd ratios [14]. The basis for the inclusion of variables in the multivariable model was a relaxed p-value of 0.2 in univariable analysis, but the evaluation of statistical significance was based on p < .05.

**Ethical considerations:** to protect the rights of the respondents, informed consent and assent were sought before data collection - which included both the guardians and adolescents. Permission to access and conduct the study in the selected schools was obtained from the County Director of Education. Additionally, ethical approval was sought and received from the University of Eastern Africa Baraton Institutional Ethics Review Committee (Approval number B132019 and renewed as UEAB/REC/02/03/2020).

## Results

**Sociodemographic characteristics of the study respondents:** most of the adolescents were female 60.3% (n= 325) while males constituted 39.4% (n= 214). The results indicated that 66.4% (n=358) of the adolescents were living with both parents, 2.4 % (n=13) of the participants reported to be living with a father only, 21.2% (n=114) were living with a mother only and 10.0% (n=54) were living with a guardian. The highest number of adolescents were Christians (77.9%) compared to Muslims (22.1%). In terms of socio-economic status, 40.1% (n=216) of the adolescents were from the middle socio-economic class, and 32.3% (n=174) said that they were from the high socio-economic class. In terms of suicide behavior, 80.0% (n=431) of the adolescents, did not report suicide risk compared to 20.0% (n=108) who reported risk of suicide. The study results indicated that 36.4% (n=196) of the adolescents had depressive symptoms while 63.6% (n=343) did not have depressive symptoms. The majority of the adolescents were not in romantic relationships (82.4%, n=444), were not sexually active (87.2%, n=470), and had never used alcohol (87.8%, n=473) and tobacco (98.9%, n=522) Table 1.

**Table 1:** socio-demographic characteristics of adolescents transitioning to public secondary schools in Nairobi County, Kenya

Variable	Category	n	%
Depression present	No	196	36.4
	Yes	343	63.6
Sex	Male	214	39.7
	Female	325	60.3
Type of school	Single	368	68.3
	Mixed	171	31.7
Religion	Christian	420	77.9
	Muslim	119	22.1
Social-economic status	Low	149	27.6
	Middle	216	40.1
	High	174	32.3
Age category	11 to 14	292	54.2
	15 to 18	247	45.8
Suicide risk	No	431	80.0
	Yes	108	20.0
Romantic relationship	No	444	82.4
	Yes	95	17.6
Bullying victimization	No	77	14.3
	Yes	462	85.7
Sexually active	No	470	87.2
	Yes	69	12.8
Lifetime alcohol use	No	473	87.8
	Yes	66	12.2
Past 30-day alcohol use	No	526	97.6
	Yes	13	2.4
Lifetime drug use	No	489	90.7
	Yes	50	9.3
Past 30-day drug use	No	527	97.8
	Yes	12	2.2
Lifetime tobacco use	No	522	96.8
	Yes	17	3.2
Past 30-day tobacco use	No	533	98.9
	Yes	6	1.1
Caregiver	Both parents	358	66.4
	Father only	13	2.4
	Mother only	114	21.2
	Guardian	54	10.0

**Risk of suicidal behavior among adolescents transitioning to secondary school in Nairobi County:** one-fifth (n=108, 20.04%) of adolescents in this study scored 7 or higher on the SBQ-R, meaning they had a high risk for suicidal behavior.

**Factors associated with risk of suicidal behavior among adolescents attending public secondary schools in Nairobi County:** in univariable analysis, depression, lifetime alcohol use, and past 30-days alcohol use were found to be significant risk factors for suicidal behavior at 95% confidence. Adolescents who reported depressive symptoms were found to have a higher prevalence ratio for suicidal behavior compared to those who did not have suicidal behavior [uPR=3.28, C.I (1.93-5.59); p=0.001]. Adolescents who had a history of lifetime alcohol use were found to have a higher prevalence ratio of suicidal behavior than adolescents who did not have a history of lifetime alcohol use [uPR=2.16 C.I (1.38-3.38); p= 0.001]. Specifically, alcohol use in the last 30 days was also a significant factor in suicidal behavior. Adolescents who had used alcohol in the past 30 days had a higher prevalence ratio for suicidal behavior than those who had not used alcohol in the past 30 days [uPR=2.38 (1.04-5.42); p=0.03]. Variables that met the relaxed threshold p-value of 0.2 at the univariable level were included in the multivariable model. In multivariable analysis, depression and lifetime alcohol use were found to be significant factors for suicidal behavior. While controlling for alcohol use, adolescents who had reported depressive symptoms had a higher prevalence ratio for suicidal behavior than those who did not report depressive symptoms (aPR=3.16, C.I {1.85, 5.41}, P=0.001). It was also found that while controlling for depressive symptoms, adolescents who had a history of lifetime alcohol use were found to have a higher prevalence ratio for suicidal behavior than adolescents who did not have a history of lifetime alcohol use (aPR=1.87, C.I {1.17, 2.97}, P=0.009) Table 2.

**Table 2:** factors associated with risk of suicidal behavior among adolescents transitioning to secondary schools in Nairobi County, poisson regression showing unadjusted and adjusted prevalence ratios

Study variables	Category	Risk of suicide				Univariable analysis			Multivariable analysis		
		No		Yes		uPR	95% CI	P-value	aPR	95% CI	P-value
		n	%	n	%						
Depression	No	180	91.8	16	8.2	Ref			Ref		
	Yes	251	73.2	92	26.8	3.28	1.93-5.59	<0.001	3.16	1.85-5.41	<0.001
Sex	Male	177	82.7	37	17.3	0.79	0.53-1.18	0.249	0.70	0.47-1.05	0.088
	Female	254	78.2	71	21.8	Ref			Ref		
Type of school	Single	295	80.2	73	19.8	Ref					
	Mixed	136	79.5	35	20.5	1.03	0.69-1.54	0.879			
Religious affiliation	Christian	332	79.0	88	21.0	1.24	0.77-2.03	0.373			
	Muslim	99	83.2	20	16.8	Ref					
Social economic status	Low	123	82.6	26	17.4	Ref					
	Middle	171	79.2	45	20.8	1.19	0.74-1.93	0.472			
	High	137	78.7	37	21.3	1.22	0.74-2.01	0.440			
Age category	11 to 14	235	80.5	57	19.5	0.95	0.65-1.38	0.771			
	15 to 18	196	79.4	51	20.6	Ref					
Romantic relationship	No	364	82.0	80	18.0	Ref					
	Yes	67	70.5	28	29.5	1.64	1.06-2.51				
Sexually active	No	379	80.6	91	19.4	Ref					
	Yes	52	75.4	17	24.6	1.27	0.76-2.14	0.362			
Bullying victimization	No	70	90.9	7	9.1						
	Yes	361	78.1	101	21.9	1.12	0.89-1.39	0.328			
Lifetime alcohol use	No	390	82.5	83	17.5	Ref			Ref		
	Yes	41	62.1	25	37.9	2.16	1.38-3.38	0.001	1.87	1.17-2.97	0.009
Past 30-day alcohol use	No	424	80.6	102	19.4	Ref					
	Yes	7	53.8	6	46.2	2.38	1.04-5.42	0.039			
Lifetime drug use	No	397	81.2	92	18.8	Ref					
	Yes	34	68.0	16	32.0	1.70	1.00-2.19	0.050			
Past 30-day drug use	No	423	80.3	104	19.7	Ref					
	Yes	8	66.7	4	33.3	1.68	0.62-4.59	0.304			
Lifetime tobacco use	No	419	80.3	103	19.7	Ref					
	Yes	12	70.6	5	29.4	1.49	0.61-3.66	0.383			
Past 30-day tobacco use	No	426	79.9	107	20.1	Ref					
	Yes	5	83.3	1	16.7	0.83	0.12-5.95	0.853			
Caregiver	Parents	295	82.4	63	17.6	0.95	0.49-1.85	0.881	0.80	0.41-1.57	0.519
	Father	8	61.5	5	38.5	2.07	0.71-6.08	0.182	1.70	0.57-5.07	0.344
	Mother	84	73.7	30	26.3	1.42	0.69-2.91	0.336	1.14	0.55-2.34	0.723
	Guardian	44	81.5	10	18.5	Ref			Ref		

CI: confidence interval, uPR: unadjusted prevalence ratios aPR: adjusted prevalence ratios

## Discussion

This study sought to elucidate the factors associated with the risk of suicidal behavior among adolescents transitioning between primary and secondary schools in Nairobi County, Kenya. This study demonstrated that lifetime alcohol use and depression are the two most important factors associated with the risk of suicidal behavior among adolescents transitioning to secondary school. These findings corroborate other similar studies which demonstrated a link between adolescent suicidal behavior and depression and alcohol use respectively [15-17]. Adolescent depression is the single most important predictor of suicidal behavior [16]. In terms of adolescent alcohol use, this current study agrees with another study which demonstrated that any lifetime use of alcohol results in a two-fold increase in the risk of suicidal behavior [17]. However, more work is needed to fully characterize and understand the magnitude and directionality of the association between alcohol use and suicidal behavior among adolescents [18]. Several authors have demonstrated an association between gender and suicidal behavior, either independently or moderating the effect of depression or other predictors [5,16,19]. However, contrary to other studies this study did not demonstrate any link between adolescent gender and suicidal behavior this could be accounted for since adolescents in this study were in the formative stages of their secondary school education, they are, arguably, a homogeneous group and the unique gender differences and challenges that they are likely to face throughout secondary school were yet to emerge. Adolescent sexual activity - especially early sexual debut or initiation - is another factor that previous studies have associated with suicidal ideation and behavior [20], as well as a moderate to high risk of suicidal behavior in Kenya [21]. Sexual activity was not shown as statistically significantly associated with the risk of suicidal behavior in this study.

This current study did not demonstrate a link between illicit drug use or other substance use and the risk of suicidal behavior among adolescents. Contrary to this, a 2019 study found that cannabis and other illicit drug use are predictors of suicidal behavior in older adolescents. Similarly, for bullying victimization - while 85.7% of adolescents had experienced some form of victimization in the two months that they had been in secondary school bullying victimization had not yet become a factor associated with suicide risk. The association of bullying victimization among adolescents with current and suicidal ideation and behavior has been demonstrated severally [22,23]. The finding that bullying victimization is not associated with suicide risk could also be explained by the fact that the victimization was still ongoing. Added to this, studies have demonstrated that the effects of victimization in adolescence are seen either in later adolescence or young adulthood [24]. The use of the cross-sectional design limits the ability to draw causal inferences in this study. However, the study findings still provide useful insights that can inform future studies on suicidal behavior among adolescents in Kenya. Our findings corroborate the findings of other studies in Africa and beyond. Therefore, our recommendations are likely applicable beyond the specific local context of our study

## Conclusion

Lifetime alcohol use and depression were associated with the risk of suicidal behavior among adolescents transitioning to secondary in Nairobi County. These factors were likely pre-existing before adolescents joined secondary schools. Therefore, preventive interventions should be targeted at primary and elementary schools. In seeking to prevent the early onset of alcohol use, the government and community stakeholders should partner to enforce alcohol distribution and consumption guidelines prohibiting the sale and consumption of alcohol to underage persons. Preventing depression among pre-secondary adolescents and children could be accomplished through contextualized school and community-based interventions to enhance social support. Additionally, while the Ministry of Health Suicide Prevention Strategy 2021-2026 is a laudable milestone, its implementation and future direction require an enhanced recognition and emphasis on adolescent suicidal behavior.

### What is known about this topic

- *Adolescents account for over 250,000 annual deaths by suicide-making suicide the second most prevalent cause of adolescent mortality around the world;*
- *Suicidal behavior in adolescents is associated with gender with females being at high risk, but male adolescents are more likely to complete suicide*.

### What this study adds

- *This study sheds light on factors associated with suicidal behavior in the adolescent transition from primary to secondary school;*
- *Depression is likely to present in pre-secondary school children and adolescents and there may be a need to intervene at the primary school level to prevent suicidal behavior;*
- *Lifetime alcohol use is a key predictor of suicide behavior in adolescents transitioning to secondary school*.
